# First Expert Elicitation of Knowledge Conducted in 2023 on Possible Drivers of Observed Increasing Epizootic Haemorrhagic Disease Incidence in Europe

**DOI:** 10.1155/vmi/5489552

**Published:** 2025-12-31

**Authors:** Claude Saegerman

**Affiliations:** ^1^ Research Unit of Epidemiology and Risk Analysis Applied to Veterinary Sciences (UREAR-ULiège), Fundamental and Applied Research for Animal and Health (FARAH) Center, Université de Liège, Liège, 4000, Belgium, ulg.ac.be

**Keywords:** clustering analysis, culicoides, drivers, epizootic hemorrhagic disease (EHD), epizootic hemorrhagic disease virus (EHDV), Europe, expert elicitation, multi-criteria decision analysis (MCDA), sensitivity analysis, uncertainty, vector-borne disease

## Abstract

Epizootic haemorrhagic disease (EHD) is a viral noncontagious arthropod‐borne disease transmitted by blood‐sucking midges of the genus Culicoides. Its causative agent, the EHD virus (EHDV), belongs to the genus Orbivirus and is responsible for domestic and wildlife ruminants’ disease outbreaks, especially in North America, Asia, Africa and Oceania. These outbreaks not only cause significant morbidity and mortality but also have welfare, social and economic implications. Recently, EHD has become a real threat to the European Union, with outbreaks confirmed in October and November 2022 in Sardinia and Sicily. To better understand this phenomenon, we investigated the drivers of the observed increasing EHD incidence in Europe through expert knowledge elicitation. We listed 51 possible drivers grouped in eight domains and elicited 41 European experts to (i) allocate a score per driver, (ii) weight this score within each domain and (iii) weight the different domains and attribute an uncertainty level for each. An overall weighted score per driver was calculated, and drivers with comparable scores were grouped in four distinct terminal nodes using a regression tree analysis. The four drivers included in a terminal node with the highest scores were (i) the influence of temperature on the abundance or survival of vector populations; the legal or illegal movements of live animals from (ii) third countries or (iii) from neighbouring countries of Europe and the European Union; and (iv) the current unavailability of vaccines against Circulating Serotype 8. Our results support researchers in prioritizing studies targeting the most relevant drivers of the observed spread of EHD in animals in Europe. In addition, some strategic lines in terms of research and action are depicted.

## 1. Introduction

Epizootic haemorrhagic disease (EHD) is a noncontagious arthropod‐borne viral disease transmitted by specific species of blood‐sucking midges of the genus Culicoides [1]. Its causative agent, the EHD virus (EHDV), has 10 double‐stranded ribonucleic acid (dsRNA) segments (seven structural [VP1–VP7] and four nonstructural [NS1, NS2, NS3/NS3A, NS4] proteins), and belongs to the family Sedoreoviridae, genus Orbivirus [2]. There are at least seven serotypes of EHDV recognized so far, with putative serotypes recently described. Ibaraki virus is the prototype member of the EHDV serogroup (Serotype 2) [2]. EHDV is transmitted by specific species of biting midges belonging to the family Ceratopogonidae, genus Culicoides, which act as biological vectors. Culicoides biting midges are hematophagous insects approximately 2–4 mm in length, which are usually active around sunrise and sunset, with activity peaks in early evening. They can bite outdoors (mostly exophilic) but also indoors (e.g., species of the Obsoletus group) when animals are housed in barns [3–6]. Larvae of many livestock‐associated species develop in warm semi‐aquatic microhabitats, especially around facilities housing ruminants [7]. For instance, according to Ref. [8], larvae of species of the Obsoletus group have been collected from dung, waterlogged soil, compost and leaf litter, while those of the Pulicaris group have generally been found in waterlogged soil.

Culicoides dispersal is described as stratified, due to the combination of dispersal processes occurring actively at short distances, and passively or semi‐actively at long distances [9]. Recent population genetics studies have demonstrated a high dispersal capacity over lands [10]. Mark‐release‐recapture studies on Culicoides species showed that the postrelease dispersal distance travelled for two nights ranges from 1 to 2.5 km and is linked to the gradual search for hosts or oviposition sites [11]. The maximum recapture distance recorded was 6 km for *Culicoides mohave* in a particular desert landscape [12]. In 2017, Sanders et al. attempted to quantify Culicoides dispersal over land and demonstrated through a capture–mark–recapture study that 84.4% of flights of more than 1 km took place downwind, while only 15.6% of flights were made upwind [13]. In addition, the dispersal happened over distances of at least 3 km. Many studies have reported a correlation between disease movement and wind‐borne transport of Culicoides during outbreaks [9, 14–18]. The introduction of bluetongue virus (BTV) serotypes by wind‐borne infected Culicoides has been demonstrated from northern Africa to southern Europe [19], from Kenya to Southwest Indian Ocean islands [20], from Sardinia to the Balearic Islands [17], from Corsica to France [18], from the south of England to Ireland [21] and from the northern coast of France and Belgium to the United Kingdom [22]. Most of these studies were supported by modelling analyses in which dispersion trajectories were assessed using overwater atmospheric dispersion models [10]. Climatic parameters considered appropriate for the survival of midges carried on wind are temperatures of 15°C–35°C, relative humidity above 25% and wind velocity of up to 10 m/sec (35 km/h) [23–26].

European Culicoides were found to be biting a wide range of mammals [27]. However, many *Culicoides* species feed preferentially on horses and domestic and wild ruminants but have been found in pig farms, and blood meal analysis demonstrates that they can feed on pigs [28, 29]. Potential vectors of EHDV in northern Europe have been shown to bite susceptible species of deer in Spain [30].

Female Culicoides of competent species become infected with EHDV and can transmit the virus to susceptible ruminants after an extrinsic incubation period of approximately 10–14 days [31]. As in the case of BTV infection, viraemia can be prolonged in EHDV‐infected ruminants because of the virus association with ruminant erythrocytes [32–34].

The incubation period of EHD in animals is estimated to be 2–10 days. The clinical signs of EHD manifest as a haemorrhagic disease in some species of deer, but domestic ruminants may also be infected [35, 36]. EHD was first described in 1955, in white‐tailed deer (*Odocoileus virginianus*) of New Jersey (USA) [37]. This species and other cervid species are severely affected by the virus, often resulting in high levels of mortality associated with high fever, lethargy, oedema, ulcerations of the dental pad and oral mucosa, haemorrhages of the heart, lungs, major blood vessels and other tissues [35]. Frequently less severe or asymptomatic and sometime mild‐to‐severe (e.g., Refs. [38–40]) EHDV infections are observed in cattle, which are considered the reservoir host for the virus [31, 41]. The clinical signs and patterns of the disease and the mode of replication of EHDV are similar to that of BTV [42, 43]. Photographs of EHD affected animals are available in the Atlas of Transboundary Animal Diseases edited by the World Organization for Animal Health (WOAH), which is intended to assist Veterinary Service field staff involved in animal disease surveillance and diagnostics to identify important transboundary diseases of livestock [43].

Economic losses due to EHD have only been properly assessed in Israeli dairy herds during an EHDV‐7 outbreak in 2006 [42]. Firstly, a high correlation was observed between EHDV seroprevalence and milk loss (average milk loss for herds with seropositivity of 26%–50%, 51%–75% and 76%–100% was 84, 133 and 204 kg of milk/milking cow, respectively), indicating the importance of active surveillance to predict the losses [38]. Secondly, a 1.42% excess mortality was observed in herds with seroprevalence above 50%. Thirdly, an average loss of USD 26.5 per cow and total losses for the dairy cattle industry ranging from USD 1,591,000 to USD 3,391,000 were estimated. Due to its economic impact in livestock, EHD was introduced as a notifiable disease to the World Organization of Animal Health in May 2008, and it is notifiable under the EU Animal Health Law, Commission Implementing Regulation 2018/1882/EU.

In North America, Asia, Africa and Oceania, EHD regularly causes large regional disease outbreaks that affect domestic and wild ruminants, mainly some species of deer, antelope and cattle. These outbreaks cause severe morbidity and mortality but also have welfare, social and economic repercussions [2, 44]. At the end of September 2021, a new strain of EHDV‐8 was reported from cattle farms in central and western Tunisia. The virus subsequently spread rapidly to the northern and eastern regions in October and November, with more than 200 confirmed outbreaks recorded [36, 45, 46]. In autumn 2022, the same virus strain was found in symptomatic cattle in both Italy (Sardinia and Sicily) and Spain (Andalusia), posing a serious threat to the European livestock industry since no vaccine is currently available [47]. During 2023, the virus spread throughout Spain, reportedly reaching Portugal on 13 July 2023 and southwest France on 4 September 2023 [48, 49].

**Figure 1:**
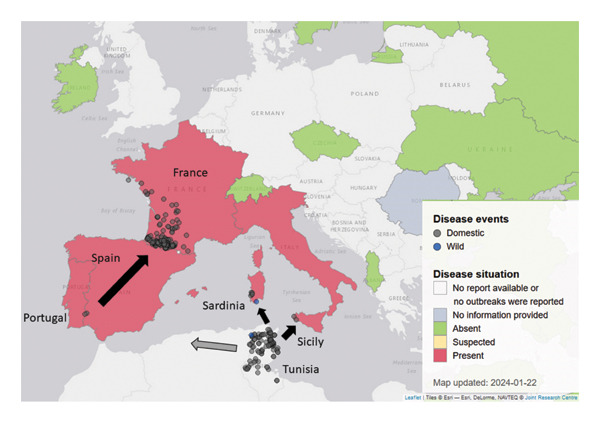
Geographical distribution of EHD confirmed outbreaks in the European Union and neighbouring countries, situation at January 2024 (source: https://animal-diseases.efsa.europa.eu/EHDV/#Geographicaldistribution). Legend: black arrows indicate the direction of spread of EHD. Grey arrow: EHDV‐8 could have circulated without notification in other western countries of the Maghreb [36, 45, 46].

Wind‐borne dispersal of Culicoides is an important process governing both the arrival of BTV into new areas and its subsequent spread [50–52]. Winds were hypothesized to be the primary cause of the long and medium distance distribution of EHDV during the 2006 Israeli epidemic [53]. It is possible that the recent and simultaneous appearance of EHDV‐8 in multiple southern territories of the EU was caused by wind flows over seawater derived from Africa’s northern region [2] (Figure 1).

The aim of this study was to investigate, for the first time, possible drivers of the observed spread of EHD in Europe, using expert elicitation of knowledge. A multicriteria decision analysis (MCDA) method was chosen because it allows the systematic integration of information from a range of sources [54] and aims to improve repeatability and transparency [55].

## 2. Materials and Methods

The process of this expert knowledge elicitation is identical to the previously published methodology utilized by the UREAR‐ULiege for other diseases that are emerging or zoonotic [56–59]. Briefly, we enumerated 51 possible drivers that were organized into eight different domains and each driver had a description that was specific to their definition (four or five modalities) (Appendix A1). Forty‐one European experts (Appendix A2) were asked to (i) rate the drivers’ capabilities, (ii) weight the rating within each domain [60] and (iii) calculate the degree to which each domain was capable of providing information and how this information was aggregated and then weighted (Appendix A3). A weighted average score per driver was determined. If all the drivers were considered equivalent by the experts, each of them would have received the same score. If not, the expert attributes more weight on the most important drivers contributing to the observed increasing EHD incidence in Europe. Finally, drivers with similar scores were clustered in several terminal nodes using a regression tree analysis (minimizing the standard error in each node). In addition, the degree of uncertainty was queried at the domain level for each expert. To identify whether the ranking of drivers of the observed increasing EHD incidence in Europe was influenced by the choice of experts or the country of origin of experts, a sensitivity analysis was performed.

The details of the methodology are described in Appendix A4 and comprising six sections: (i) the questionnaire design; (ii) the expert elicitation to assess the drivers of observed increasing cases of EHD in Europe; (iii) the scoring, weighting system and level of uncertainty; (iv) the calculation of an overall weighted score for each driver and ranking process; (v) the cluster analysis and (vi) the sensitivity analysis to test the robustness of the expert elicitation.

## 3. Results

### 3.1. Response Rate and Field of Expertise Mobilized by the Experts

Sixty‐eight professionals with scientific knowledge and/or field knowledge or experience regarding EHD and EHDV were contacted, i.e., respectively, 16, 20 and 32 from Belgium (boundaries shared with France that experienced EHD outbreaks), France (country currently with cases of EHD) and other European countries concerned by the future EHD spread. The fields and diversity of expertise are summarized in Appendix A2. Among all professionals contacted, 13, 12 and 16 responded and originated from Belgium, France or other European countries, respectively. The participation rate was 60.3%.

### 3.2. Estimating the Overall Weighted Score and Ranking of the Observed Increasing EHD Incidence in Europe

**Figure 2:**
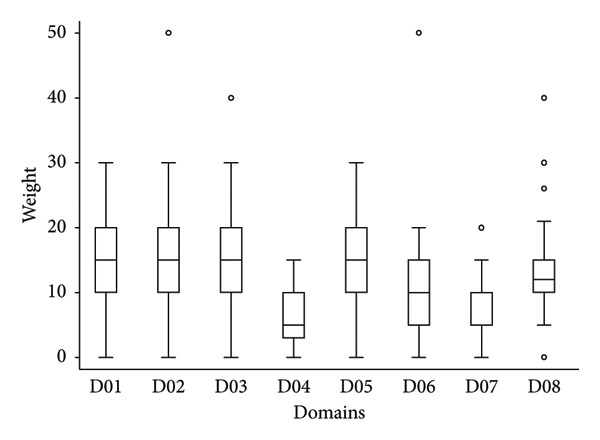
Boxplot of the relative importance of the eight domains of possible drivers of the observed increasing cases of EHD in Europe (*N* = 41 European experts). Legend: The bold line represents the median of the score distribution between the different experts attributed to each domain; the solid lines at the bottom and top of each rectangle represent, respectively, the first and the third quartiles; adjacent lines to the whiskers represent the limits of the 95% confidence interval; small circles represent outside values. The eight domains of drivers are D01, disease/pathogen characteristics; D02, distance to Europe and the expert’s country (spatial–temporal scales); D03, ability to monitor, treat and control the disease; D04, characteristics of European farms; D05, global change; D06, interface with wildlife; D07, human activity; and D08, economic and trade activities.

The medians of the weight between the eight domains of drivers as well as for the different 51 drivers were not equal according to the nonparametric Kruskal–Wallis test (chi‐squared test = 71.5 with 7 d.*f*. and *α* = 0.05, *p* value = 0.0001; and chi‐squared test = 744.2 with 50 d.*f*. and *α* = 0.05, *p* value = 0.0001, for the weights between domains and weights of the different drivers, respectively) (Figure 2). The median of the weights of domains D04 (*p* value < 0.001), D06 (*p* value = 0.034) and D07 (*p* value < 0.001) were significantly lower than the median of D01 as a reference (median regression).

**Figure 3:**
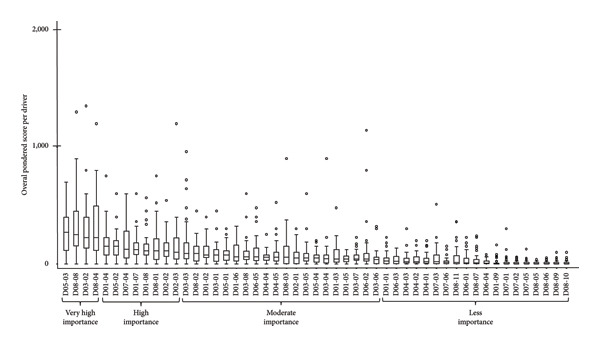
Ranking of the overall weighted score for each potential driver of the observed increasing epizootic haemorrhagic disease incidence in Europe (boxplot based on input from 41 European experts). Legend: *X* axis represents the drivers with the following codification: D01–D08 refer to the eight domains of drivers and D01–01 to D08–11 refer to a specific driver (for the codification, see Appendix A1). Drivers were grouped as having ‘very high importance’, ‘high importance’, ‘moderate importance’ and ‘less importance’ according to the cluster analysis of their weighted scores.

Twelve drivers out of 51 were ranked in decreasing order as being of very high importance (*N* = 4) and of high importance (*N* = 8) in the probability of playing a key role in the observed increasing EHD incidence in Europe. The first most important driver was from the global change domain, the influence of the temperature on the abundance/survival of the vector populations (D05–03). The second and the fourth most important drivers were from the economic and trade activities domain, the legal or illegal movements of live animals from third countries (i.e., countries that are not a member of the European Union as well as countries or territories whose citizens do not enjoy the European Union right to free movement) (D08–08) and from neighbouring or European Union countries (D08–04), respectively. The third most important driver was vaccine availability (D03–02). The following eight drivers, with high importance in the probability of playing a key role, were the ease or speed of the pathogen spread (D01–04); the influence of humidity on the abundance or survival of the vector population (D05–02); live animal transport vehicles (D07–04); the existence of vectors and potential spread (D01–07), the mode of transmission of the pathogen, i.e., via vector (D01–08); the decrease in resources allocated to disease surveillance in animals and/or in the environment (D08–01); the European geographic proximity of the pathogen or disease to the expert’s country (D02–02); and recently reported cases in Europe (D02–03) (Figure 3).

### 3.3. Cluster Analysis

**Figure 4:**
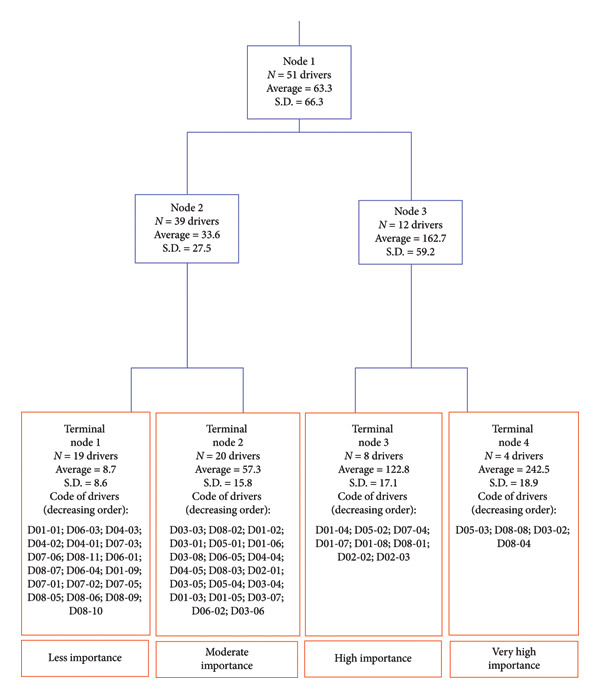
Aggregation of drivers of the observed increasing epizootic haemorrhagic disease incidence in Europe into four homogenous groups using a regression tree analysis. Legend: *N* = Number, S.D. = Standard deviation.

Results of the regression tree analysis revealed four significantly different clusters of drivers (Figure 4) according to the nonparametric Kruskal–Wallis test (chi‐squared test = 44.2 with three degrees of freedom (d.f.) and *α* = 0.05; *p* value = 0.0001). Four drivers were assigned the category of ‘very high importance’, eight drivers received the category of ‘high importance’, twenty drivers received the category of ‘moderate importance’ and 19 drivers received the category of ‘less importance’.

### 3.4. Sensitivity Analysis of the Impact of Experts on the Final Ranking of the Observed Increasing EHD Incidence in Europe

Firstly, the results of two different sensitivity analyses indicated that, regardless of the expert excluded, removing some experts only had very limited and no significant effects on the ranking compared to the reference (all experts elicited). Secondly, using 10 bootstraps of 30 experts among 41, the Pearson correlation coefficient between bootstraps against the ranking of 41 experts as a reference was very high (value between 0.974 and 0.994, with a *p* value of < 0.0001). Thirdly, when comparing the ranking of drivers between either Belgian experts (*N* = 13), French experts (*N* = 12) or other experts (*N* = 16) and all experts as a reference, the Pearson coefficient of correlation was of 0.952, 0.974 and 0.970, respectively, with a *p* value of < 0.0001.

### 3.5. Level of Uncertainty per Domain of Drivers

**Figure 5:**
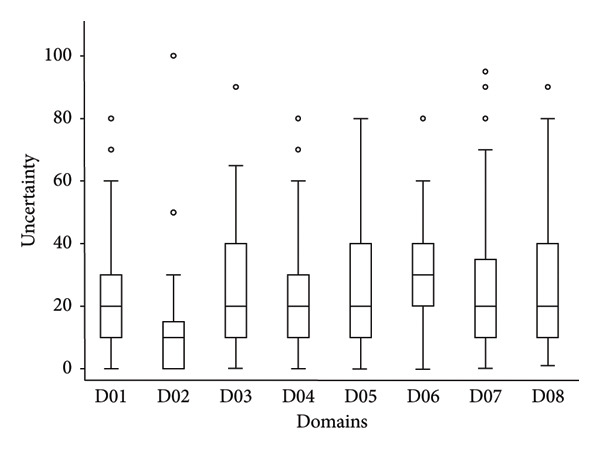
Level of uncertainty per domain of drivers. Legend: The bold line represents the median of the level of uncertainty attributed by experts using a scale from 0 (minimal uncertainty in the scoring) to 100 (maximum uncertainty in the scoring); the solid lines at the top and bottom of each rectangle represent, respectively, the first and the third quartiles; adjacent lines to the whiskers represent the limits of the 95% confidence interval; and small circles represent outside values. The eight domains of drivers are D01, disease/pathogen characteristics; D02, distance to Europe and the expert’s country (spatial–temporal scales); D03, ability to monitor, treat and control the disease; D04, characteristics of the European farms; D05, global change; D06, interface with wildlife; D07, human activity; and D08, economic and trade activities.

The level of uncertainty per domain of drivers is shown in Figure 5. The medians of the uncertainty between domains of drivers were not equal according to the nonparametric Kruskal–Wallis test (chi‐squared test = 32.5 with 7 d.*f*. and *α* = 0.05, *p* value = 0.0001). Domain D02 (distance to Europe and the expert’s country) had a median uncertainty of 10 and Domain D06 (interface with wildlife) had a median uncertainty of 30, respectively, significantly higher and lower than the median of the other domains, which was equal to 20 (median regression; *p* value = 0.05). In addition, there is no linear (Pearson coefficient correlation = −0.09; *p* value = 0.11) or nonparametric (Spearman rank correlation = −0.08; *p* value = 0.13) relationship between the median weight and the median uncertainty per domain.

## 4. Discussion

Fifty‐one possible drivers of observed increasing EHD incidence in Europe were ranked and aggregated into four homogenous groups according to expert elicitation of knowledge. Only the first 12 most important ranked drivers will be further discussed with a focus on those categorized in the regression tree as ‘very high importance’ (*N* = 4) and ‘high importance’ (*N* = 8), respectively. Moreover, the sensitivity analysis showed a limited and insignificant effect of experts involved (using both bootstraps and country‐origin groups of experts), indicating an acceptable robustness of the expert elicitation for drivers included in the first two terminal nodes of the tree. In addition, the median‐level of uncertainty for all domains of drivers was moderate (around 20 on a scale from 0 up to 100) except for Domain D02 (distance to Europe and the country of expert—spatial–temporal scales), which was less (around 10), and Domain D06 (wildlife interface), which was higher (around 30). Regarding the last domain with the highest median uncertainly, more surveillance and research‐action are recommended on European wildlife to assess more properly the susceptibility of deer species regarding to EHDV‐8 and its possible role as the reservoir of the disease [2, 34, 61].

The most important driver for the observed increasing cases of EHD in Europe recognized during this expert elicitation was the influence of the temperature on the abundance or survival of vector populations (D05–03). In Sardinia, EHDV‐8 was detected in whole bodies of *C. imicola*, *C. obsoletus*/*C. scoticus*, *C. newsteadi*, *C. pulicaris* ss and *C. bysta*. Note that simple detection of RNA of EDHV is not the proof of vector competency and capacity. However, since the first four species are also able to transmit BTV, and given the current distribution of BTV in Europe, further spread of EHDV‐8 is expected in Europe [62]. According to a recent modelling study, the habitat suitability of *C. imicola* will likely expand to higher latitudes due to three main variables identified in decreasing order of importance, i.e., the temperature seasonality, the precipitation of the coldest quarter and the average temperature of the wettest quarter with a 30.3%, 29.5% and 16.5% contribution, respectively [63]. This modelling confirms earlier studies (e.g., Refs. [52, 64]). Indeed, the current suitable habitats for the Afro‐Asian biting midge vector *C. imicola* are distributed in most of the southern part areas of America, southwestern Europe, most of Africa, the coastal areas of the Middle East, almost all regions of South Asia, southern China, a few countries in Southeast Asia and the whole of Australia [63]. Currently, we do not know that EHDV‐8 can be vectored by Palaearctic species of Culicoides but expects northwards. For example, in France, EHDV‐8 has already spread in areas where *C. imicola* is absent.

Under experimental conditions, the temperature also has a significant impact on the spring emergence of two Palaearctic species of Culicoides, *C. chiopterus* and *C. dewulfi* [65]. In this experiment, the emergence of Culicoides started when the temperature exceeded 20°C for several days [65]. In contrast, Losson et al. [66] recorded a few Culicoides captured during the 2006–2007 winter in Belgian cattle shed, at minimum temperatures of between 6°C and 12°C. Subsequent studies have confirmed the trapping of Palaearctic species of Culicoides at similar or even slightly lower minimum temperatures [67, 68]. Another study demonstrated activity down to 4°C [69].

The temperature has a crucial effect on the extrinsic incubation period, i.e., the time between uptake of virus by the insect vector and its presence in the salivary gland. However, due to the nature of the required experimental work, temperature thresholds have not been systematically investigated for EHDV at this time [70]. Temperature also influences biting rates [71]. Temperature is undoubtedly the main environmental factor influencing the behaviour and survival of these midges; their activity peaks between 13°C and 35°C although these limits vary according to species [72]. In relation to BTV, Culicoides kept at 15°C require several weeks to complete the extrinsic incubation period, while those kept at 30°C may complete incubation in a couple of days [73]. Higher temperatures can reduce the time needed for a vector to digest a blood meal, increasing the frequency of blood‐feeding [8]. All these events increase the opportunities for transmission [8].

**Figure 6:**
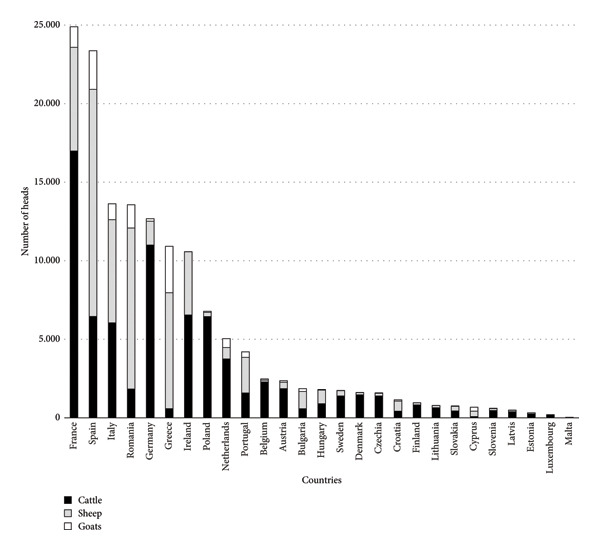
Ruminant population by EU Member States, expressed in thousand heads (animals) (source: Ref. [77]).

In Europe, since June 2023, every month has been the warmest on record for the respective month of the year [74]. There is likely to be an effect of such climate pressure on the abundance, activity and survival of Culicoides and their vector competence. The distribution (map) of different Culicoides species in Europe is frequently updated on the dedicated website of the European Centre for Disease Prevention and Control (ECDC) and European Food Safety Authority (EFSA) at the following address https://www.ecdc.europa.eu/en/disease-vectors/surveillance-and-disease-data/biting-midge-maps [75]. In the European context, reported EHD outbreaks are more numerous in domestic than in wildlife ruminants (Table 1). This is perhaps because wild ruminant EHD outbreaks are underreported, not because of a lower incidence or impact. In fact, in Europe, mandatory EHD surveillance is based on clinical signs. As mortality may be low at the individual level (one or a few cases by herd) and as wildlife surveillance is more often based on cadaver observation, a surveillance system for wildlife based on clinical signs would likely result in unobserved cases of EHD. In addition, the size of domestic and wild ruminant populations and their connectivity are of prime importance to understanding the current spread of EHD (Figure 6). To better assess the role of wildlife as the possible reservoir of EHDV‐8, we recommend more active surveillance of wildlife.

**Table 1:** Confirmed epizootic haemorrhagic disease outbreaks officially notified at the European level since its first discovery, situation at the end of 2023.

Country	First event	Last event	Domestic ruminants			Wild ruminants^(a)^		
			Cattle	Sheep and goats	Domestic deer	Reed deer (*Cervus elaphus*)	Roe deer (*Capreolus capreolus*)	Fallow deer (*Dama dama*)
Spain	15/11/22	15/11/23	251^(b)^	0	20^(d)^	?^(b)^	1	0
France	04/09/23	17/11/23	3764^(c)^	0	0	0	0	0
Italy (Sardinia)	28/10/22	19/10/23	8^(e)^	1^(e)^	0	1	0	1
Italy (Sicily)	25/10/22	09/12/22	2	0	0	0	0	0
Portugal	13/07/23	13/09/23	71	0	2	0	0	0
Total	25/10/22	07/11/23	4096	1	22	1	1	1

Source: Refs. [49, 76].

^(a)^Some other cases may be known to wildlife monitoring services.

^(b)^Instead of counting the total number of outbreaks, Spain registers the first case by each local veterinary unit. Outbreaks in cattle and in red deer are not differentiated in the figures provided in 2023 (https://www.mapa.gob.es/es/ganaderia/temas/sanidad-animal-higiene-ganadera/notaehe_30_11_2023_tcm30-667555.pdf).

^(c)^Ministry of Agriculture and Food Sovereignty—situation at 18/01/2024 (https://agriculture.gouv.fr/mhe-la-maladie-hemorragique-epizootique). Twenty departments in South‐West France are affected by these outbreaks (Pyrénées‐Atlantiques, Hautes‐Pyrénées, Haute‐Garonne, Gers, Landes, Ariége, Aude, Tarn, Lot‐et‐Garonne, Gironde, Tarn‐et‐Garonne, Dordogne, Corrèze, Vendée, Deux Sévres, Loire‐Atlantique, Lot, Haute‐Vienne, Morbihan, and Pyrénées‐Orientales). Note that in southwestern France the wildlife ungulates are also well represented.

^(d)^The density of animals is generally higher in domestic deer compared to wildlife.

^(e)^Bovine of eight holdings showed clinical signs; sheep in one farm only showed positive PCR and seroconversion (seroneutralisation and ELISA).

The second and the fourth most important drivers were related to the (il‐)legal movements of live animals from third countries (D08–08) or neighbouring countries of Europe and the European Union (D08–04). In European countries, there are currently fewer movements of live domestic animals originating from third countries than from neighbouring countries or European Union Member States (MS) (see also international trade statistics, available at the following URL address: https://www.trademap.org/tradestat/Index.aspx; accessed on 29 December 2023). Nevertheless, a proper estimation of the relative importance of illegal movements of live animals and their introduction pathways is deemed essential to set up risk‐based awareness, prevention and surveillance programs that correspond to reality [57]. A previous EFSA assessment of the risk of introduction of EHDV into the European Union was developed by considering three possible entrance pathways: (i) via imported infectious animals and (ii) via infectious vectors and (iii) other routes, e.g., via vaccines or germplasm. When quarantine and testing for EHDV are in place, the probability of importing an infectious animal into an EU MS is considered negligible. However, the probability of introducing an infectious animal through illegal livestock movements, illegal wild animal movements or wildlife transit could be high depending on the origin (EHDV circulating area) and the season of movement (vector activity). Indeed, following the introduction of EHDV by an infectious animal, the risk of exposure of EU susceptible animals during periods of vector activity was considered high. Depending on the temperature at the time of introduction (see also the main driver D05–03), the risk of spread was considered moderate or high [78].

The third most important driver was related to the vaccine availability for animals (D03–02). The only commercially available and currently used vaccines are manufactured for EHDV‐2 in Japan and are either live or inactivated vaccines [79]. In addition, there are currently no licenced vaccines available for EHD in the United States (for white‐tailed deer) other than autogenous vaccines [80], but some autogenous vaccines do not result in a high titre for homologous virus serotypes [81]. Currently, in Europe and Tunisia, the same EHDV Serotype 8 circulates with very high nucleotide sequence identity [46, 47] and no specific vaccine exists [44]. Some isolates of EHDV‐6 and EHDV‐8 previously showed some cross‐reactivity [44]. To limit EHDV infections, stop the virus from spreading and stop direct and indirect economic losses, more research is required on the development of vaccines [47, 80]. To encourage vaccine development, an assessment of the ratio of vaccine costs to EHD expenses may be beneficial (e.g., for a basic methodology [82]). If a vaccine is produced, its use could be regulated under the temporary authorization of animal vaccinations that have been previously discussed [83]. Several conditions must be fulfilled before a temporary authorization can be granted, e.g., ‘inactivated vaccines should be used preferentially in order to exclude reversion to virulence and re‐assortment between vaccine viruses and/or field strains of EHDV (and possibly a Differentiating Infected from Vaccinated Animals (DIVA) strategy would be great); decision‐making must be supported by scientific evidence and risk analysis; there must be a complete census of the susceptible animals that were vaccinated; vaccination protocols must be adhered to and there must be a scheme allowing for registration, delivery and follow‐up of vaccination, and monitoring, analysis and, possibly, adjustment of field use of the vaccination’ [83]. This temporary authorization must be replaced by a full authorization as quickly as possible. However, if the circulation of multiple serotypes could occur in the same area, farmers may be reluctant to vaccination against EHDV.

Three drivers were connected, i.e., the fifth (the ease or speed of spread of the pathogen; D01–04), the eighth (the existence of the vectors and potential spread; D01–07) and the ninth (the vector‐borne mode of transmission of the pathogen; D01–08) most important, according to the weighted scores. The basic reproduction ratio (*R*_0_) is currently not known for EHD under European conditions but was estimated as moderate [84]. However, EHD is a ‘bluetongue‐like’ disease of ruminants caused by EHDV, which is also a Culicoides‐transmitted Orbivirus very closely related to BTV [85]. Using the surrogate data of the past BTV‐8 epizootic [86], the median *R*_0_ of EHD can be provisionally estimated to be around 2. The speed of EHDV spread is currently undetermined and depends on the environmental heterogeneity and the presence of susceptible species (Figure 6), but the velocity has been estimated at ranging from 7.9 km/week (BTV‐1 in 2014 in Italy) to 24.4 km/week (BTV‐1 in 2008 in France) [87], up to 39 km/week (BTV‐8 in 2007–2008 in France) [88]. In addition, despite the restriction on animal movements, the effect of vaccination with inactivated vaccine on BTV speed was estimated (BTV‐1 in 2008 in southwestern France) at 3.2 km/day, i.e., a decrease from 5.4 km/day in nonvaccinating municipalities to 1.7 km/day in municipalities with vaccination [89]. The current observed spatiotemporal spread of EHDV‐8 in 2023 in France illustrates well the previous considerations and the competency and capacity of European Culicoides species.

The sixth most important driver was the influence of humidity on the abundance or survival of the vector population (D05–02). A > 25% relative humidity is a climatic parameter considered appropriate for the survival of midges [23–26]. A recent modelling study has indicated that the precipitation in the coldest quarter is an important predictor of the habitat suitability for *C. imicola* [63]. Indeed, the larval development of most Culicoides species is optimal in semi‐aquatic microhabitats, consisting mainly of warm, moist or wet substrates, rich in organic matter [90, 91]. In general, the larvae are mainly found in the first 5‐6 cm of the upper layer of the environmental substrate [92]. The pupae are found on the surface of the substrate (mud or water) where larval development takes place [91]. In terms of abiotic factors, temperature, humidity and rainfall are of importance for the vector biology, but temperature seems to be the most important parameter that was illustrated by this expert elicitation and supported by the scientific literature under field and experimental conditions (e.g., Refs. [93, 94]).

The seventh most important driver was live animal transport vehicles (D07–04). As an example, the intra‐EU trade of live animals in 2019, for several reasons, such as slaughter, fattening or breeding, represented around 4.18 million cattle and 3.36 million sheep and goats. For the same year, the extra‐EU trade of life animals represented 3.04 million cattle and 0.98 million sheep, mostly for exportation. Animals are transported primarily by sea and by road [95]. Whatever the direction (export or import), the risk of EHDV infection exists if *Culicoides* spp. are present in the live animal transport vehicle. Such a risk has not been estimated for EHDV so far. However, a previous risk assessment model has been built to assess the probability of a BTV‐8 outbreak following the introduction of Culicoides via live animal transport vehicles. The mean weighted annual risk for an outbreak caused by the transportation of a single vector from an affected northern European country varied from 1.8 × 10^−7^ to 3.0 × 10^−13^, depending on the country of origin [96]. Indeed, this mechanism represents a significant risk to BTV‐free countries, if a large number of vectors are transported. To date, there is no conclusive evidence that the use of insecticides or repellents singularly reduces the transmission of BTV in the field [97]. In specific scenarios, however, they have been shown to either kill biting midges or reduce host/vector contacts, and hence, they are used as mitigation when vaccines are not available [97], which is the case for EHDV‐8 in Europe at this time. Stabling is also effective in reducing host/vector contact where a high level of containment can be attained [97], but its feasibility under field conditions is of low. Insecticide‐treated meshes settled over stables windows in stables were found to kill biting midges quickly enough to prevent entry; field trials demonstrated substantial reductions in populations found in stables [98, 99]. As these studies mostly focused on horses, the logistics and reduced coverage existing in ruminant facilities may lessen such effects [97]. However, these measures could be applicable to the protection of animals from Culicoides during transport [99]. In addition, the chemical compounds derived from the neem tree (*Azadirachta indica* A. Juss; Meliaceae) have shown antilanding and antifeeding effects on adults of a colony of *C. nubeculosus* and field‐collected *C. impunctatus* [100]. Neem cake is a cheap and ecofriendly by‐product obtained from the extraction of neem oil and was used in a field case study (randomized complete block design with four replications of the treated and untreated plots) in Sardinia, Italy [101]. In this study, a treatment with neem cake at a dose of 100 g/m^2^ was applied on a larval breeding site of Culicoides located on the edge of a pond in a livestock farm. The emergence of Culicoides adults in treated and untreated plots was observed weekly using emergence traps before and after treatment. In neem cake‐treated plots, a significant reduction in the emergence of Culicoides was recorded up to 28 days. *C. imicola* represented about 10% of all emerged Culicoides adults and were highly sensitive to the neem cake [102]. Further studies are needed to gain more lines of evidence and design appropriate standard operational procedures to secure animal transportation. Furthermore, it is unclear what potential risk of introduction from EHDV‐infected midges exists during transport by airports, ships, trains or ports from countries that are not EHDV‐free. The closeness of the first outbreaks of BTV‐8 in 2006 and BTV‐3 in 2023 to international airports in Belgium and the Netherlands is striking. This raises the question of whether animal‐free transport of fresh products such as fruit or flowers from EHDV‐positive third countries generates a certain risk of EHDV strains entering Central Europe.

The 10th most important driver was the decrease in resources allocated to disease surveillance in animals and/or in the environment (D08–01). The resource attribution at EU level is related to the first pillar of the Animal Health Strategy with the objectives to (i) progressively eliminate animal diseases and to implement disease monitoring measures in the MS and the EU as a whole; (ii) ensure a high level of animal health, public health and consumer protection; and (iii) guarantee a high level of protection of both animal health and public health, to encourage the improvement of the productivity of the livestock sector and to contribute to the economic sustainability of the sectors directly or indirectly affected by an animal disease outbreak [103]. This is a challenging equilibrium especially in the context of recent major events (COVID‐19 and a resurgence of conflict and violence) [104, 105]. For BTV, a decrease of resources allocated to the disease surveillance in animals was observed from 112 million euros in 2009 to 3.8 million euros in 2014 [103]. Sufficient allocation is needed, especially during this winter to ensure a representative picture of the presence of EHDV occurrence in vector and domestic and wildlife compartments using both clinical and active surveillance (serological and molecular testing as the clinical expression of the disease can be mild or asymptomatic) when considering the current spread.

The 11th and the 12th most important drivers were the European geographic proximity of the pathogen/disease to the expert’s country (D02–02) and the recently reported cases in Europe (D02–03). It is important to be aware of the EHD situation and circulating serotype(s) of EHDV close to one’s country and to prepare some mitigation measures in the at‐risk countries. EHD is categorized as D and E under the EU Animal Health Law (Regulation [EU] 2016/429), which impose an obligation for livestock farmers to monitor for the disease and notify outbreaks, as well as restrictions on movements in affected MS. The EHD situation is registered in the EU Animal Diseases Information System (ADIS: https://food.ec.europa.eu/animals/animal-diseases/animal-disease-information-system-adis_en). In addition, the current incursion of EHD in southern Europe demonstrates the need to further promote a ‘global health’ approach, i.e., a long‐term collaboration between south and north countries to anticipate emerging vector‐borne diseases in the context of climate change. In this context, investment in capacity building (e.g., twinning programs for laboratories) and surveillance systems in the south can help northern countries to better anticipate any new event.

Expert knowledge elicitation may be limited by factors such as cognitive bias, overconfidence or replies that are influenced by recent, well‐publicized research (for a review, see Ref. [103]). The standardization of this expert elicitation (e.g., number of experts, choice of experts, different disciplines involved, protocol followed) and its sensitivity analysis takes partially these limitations into consideration in this elicitation to some extent. Furthermore, expert knowledge elicitation is an addition to empirical research, not a replacement for it [59]. Expert elicitation can be quickly implemented and empirical research that follows transparent and well‐designed protocol needs time. Both are necessary and not in opposition but should be a continuum with iterative process. In addition, as reviewed by prominent scientific journals, the elicitation of scientific and technical judgements from experts, in the form of subjective probability distributions, can be a valuable addition to other forms of evidence in support of public policy decision‐making [106].

The findings are specific to the European context (the purpose of this paper), but well the methodology can be easily translated to other regions and diseases (e.g., Refs. [56–59]).

## 5. Conclusions

Scientific knowledge on possible drivers of observed increasing animal cases of EHD in Europe was scanned. In this context, expert elicitation of knowledge and MCDA, in addition with clustering and sensitivity analyses together, allowed the identification of 12 drivers of either very high (*N* = 4) or high importance (*N* = 8), on which to concentrate future studies in order to increase and refine the understanding of the epidemiology of EHD under specific conditions of Europe and to support decision‐making to reduce exposure to EHDV and its impacts.

This expert elicitation of knowledge identified three main strategic axes of research or action. The first axis is the need for more research development to find a safe and protective vaccine towards EHDV‐8, possibly within the framework of a temporary authorization to vaccinate animals in emergency situations. The second axis is the promotion and operationalization of the ‘global health’ to anticipate the emergence of new pathogens or vectors in Europe, such as zoonotic arboviruses, which are already circulating in southern regions. This may be facilitated by the import of both new vectors and existing vectors attaining new competency capabilities to these new pathogens, in particular, due to climate change (e.g., Ref. [107]). The third axis includes awareness and information campaigns targeting both farmers and practitioners, strategies for the detection of infected animals (including subclinical presentations), systematic epidemiological investigations including vector surveillance and both passive and active surveillance of both susceptible ruminants and wildlife animal populations (especially at the front of EHD spread), restriction of movements and efforts to reduce contact between hosts and vectors [2, 108, 109].

## Conflicts of Interest

The author declares no conflicts of interest.

## Author Contributions

Conceptualization, methodology, software, validation, formal analysis, investigation, resources, data curation, writing–original draft preparation and writing–review and editing: Claude Saegerman; Experts included in the expert elicitation of knowledge are listed in Appendix A2.

## Funding

No funding was received for this manuscript.

## Data Availability

The data that support the findings of this study are available from the corresponding author upon reasonable request.

## Appendix A1: Domains of Each Defined Driver and Their Respective Defined Coefficients (Scores).

Table A1

**Table A1:** Domain D1. Disease/pathogen characteristics.

D1‐1	*Current knowledge of the pathogen (e.g., transmission routes, incubation period depending of the transmission route and infectious dose)*	
	Score 0	
	Score 1	Very high: deep scientific knowledge on the pathogen, extensive scientific literature available on its biology (transmission mode, infectivity, etc.).
	Score 2	High: detailed scientific knowledge on the pathogen but conflicting scientific results; some elements of the pathogen’s biology are still not elucidated.
	Score 3	Moderate: limited scientific knowledge on the pathogen agent because it is still under characterization; pathogen recently discovered/isolated but belonging to a well‐known and studied family of pathogens; the pathogen is characterized by multiple variants not characterized yet.
	Score 4	Low: lack of scientific knowledge on the pathogen (multiplication, infectivity, incubation period, transmission mode, transmission route, etc.); pathogen agent recently discovered and emerging;

D1‐2	*The current species specificity of the disease-causing pathogen*	
	Score 0	
	Score 1	Low: only one species is involved.
	Score 2	Medium: two species involved.
	Score 3	High: three species involved.
	Score 4	Very high: more than three species involved.

D1‐3	*Genetic variability with time of the infectious agent*	
	Score 0	Negligible: the infectious agent is genetically stable.
	Score 1	Low: the genetic variability is low; therefore, it has a low effect in the (re)emergence of the pathogen.
	Score 2	Medium: the pathogen can be considered with a medium genetic variability.
	Score 3	High: the pathogen is considered with a high genetic variability.
	Score 4	Very high: very high genetic variability (e.g., high mutation rate, re‐assortment and recombination).

D1‐4	*Transmission of the pathogen in relation to the possible spread of the epizootic (i.e., ease/speed of spread)*	
	Score 0	
	Score 1	Low: low and slow transmission within farms. For example, between farms only if an infected animal is introduced, close contact.
	Score 2	Medium: medium ease/speed transmission within the farm and between farms.
	Score 3	High: fast transmission within a farm. In a short period of time, all animals of the farm are infected. Adjacent farms become infected fast.
	Score 4	Very high: very fast and high transmission within the farms and between farms. A complete area is infected in a very short period of time.

D1‐5	*Risk of showing no clinical signs and silent spread during infection and postinfection in animals*	
	Score 0	Null: silent spread is not part of the pathogen’s characteristics.
	Score 1	Low: very short incubation period and signs of infections easily detected/recognized.
	Score 2	Moderate: very short incubation period and signs of infection are not easily detected/recognized.
	Score 3	Medium: long incubation period, clinical signs are not characteristics, and therefore, specific diagnosis is necessary to detect infection.
	Score 4	Very high: long incubation period. Disease/infection shows no clinical symptoms during the infectious period. Chronic shedder.

D1‐6	*The number of wildlife compartments/groups (e.g., ungulates) playing a role as reservoir or amplifying host for the pathogen and potential spread from it*	
	Score 0	Null: no known wildlife reservoir. The pathogen has never been reported in wildlife species (i.e., possible detection but not necessary proof of reservoir or amplification host).
	Score 1	Low: the pathogen has been reported in only one group of wildlife.
	Score 2	Moderate: the pathogen has been reported in two groups of wildlife.
	Score 3	High: the pathogen has been reported in at least three groups of wildlife.
	Score 4	Very high: disease establishes itself in wildlife as a reservoir and very hard to eradicate it from wildlife. Livestock easily infected through contact with wildlife. OR the pathogen has never been investigated in wildlife species.

D1‐7	*Existence of vectors (e.g., mosquitoes, midges, Culicoides) and potential spread.*	
	Score 0	Null: no known other vector.
	Score 1	Low: only one type of vector is present in the country, but its role in the transmission is presumed low (has not been assessed to date).
	Score 2	Moderate: only one type of vector exists in the country and has only been suspected as the source of disease spread.
	Score 3	High: only one vector is present in the country and can carry and spread the pathogen.
	Score 4	Very high: more than one type of vector can carry and spread the pathogen and are spread in most of the territory.

D1‐8	*Transmission of the pathogen to animals*	
	Score 0	
	Score 1	Low: animals are infected by direct close contact with other infected animals and by vertical transmission.
	Score 2	Moderate: transmission by direct and indirect contacts only (e.g., through vehicles, clothes, instruments) or nonflying arthropod vector (e.g., ticks).
	Score 3	High: vector transmission by flying vectors (e.g., *Culicoides*).
	Score 4	Very high: more than three modes of transmission and/or airborne transmission.

D1‐9	*Environmental persistence*	
	Score 0	Null: pathogen does not survive in the environment.
	Score 1	Low: only anecdotal isolation of the pathogen from the environment has been recorded.
	Score 2	Moderate: the survival of the agent in the environment is limited (only temporary) and it is dependent on certain environmental conditions such as humidity, temperature and rainfall.
	Score 3	High: the survival of the agent in the environment is limited (only temporary) and not dependent on certain environmental conditions such as humidity, temperature and rainfall.
	Score 4	Very high: agent naturally surviving in the environment (soil and water) and organic materials where it has long‐term survival.

*Note:* Number of drivers = 9, hence 90 points to be distributed within this domain for the intradomain weighting.

Table A2

**Table A2:** Domain D2. Distance to Europe and your country.

D2‐1	*Current incidence (cases)/prevalence of the disease in the world excluding Europe (the case of Europe is considered in the next criterion)*	
	Score 0	
	Score 1	Low: disease reported only in the countries of the Australasia region (Australia, New Zealand, New Guinea and neighbouring Pacific Islands).
	Score 2	Moderate: disease reported in countries of the Americas, Caribbean and Asia (excluding the Russian Federation).
	Score 3	High: disease was reported/present in Africa.
	Score 4	Very high: disease was reported in countries of the Mediterranean Basin, Middle East or the Russian Federation.

D2‐2	*European geographic proximity of the pathogen/disease to your country*	
	Score 0	Very low: disease never been present in Europe.
	Score 1	Low: disease reported in Europe in the past but is currently exotic.
	Score 2	Moderate: disease currently present in at least one European country that does not border your country.
	Score 3	High: disease currently present in at least one of the countries bordering your country.
	Score 4	Very high: disease reported in your country (cases and/or infections in animals).

D2‐3	*To your knowledge when was the disease last reported in Europe*	
	Score 0	Negligible: more than 20 years ago.
	Score 1	Low: more than 10 years ago.
	Score 2	Moderate: more than 5 years ago.
	Score 3	High: more than 1 year ago.
	Score 4	Very high: currently present in Europe.

*Note:* Number of drivers = 3, hence 30 points to be distributed within this domain for the intradomain weighting.

Table A3

**Table A3:** Domain D3. Ability to monitor, treat and control the disease.

D3‐1	*Ability of preventive/control measures to stop the disease from entering the country or spreading (containment of the epidemic), excluding treatment, vaccination and vector(s)/reservoir(s) control*	
	Score 0	
	Score 1	Very high: sanitary certificate; effective traceability of animals and by‐products; effective disinfection measures; no contact between domestic and wild animals; effective biosecurity measures.
	Score 2	High: no sanitary certificate; effective traceability of animals and by‐products; effective disinfection measures; limited or incomplete possibilities to restrict contacts between domestic and wild animals; effective biosecurity measures.
	Score 3	Low: no sanitary certificate; incomplete traceability of animals and by‐products; ineffective disinfection measures; incomplete restriction of contacts between domestic and wild animals; incomplete restriction of wildlife movements; ineffective biosecurity measures.
	Score 4	Very low: no sanitary certificate; no traceability of animals and by‐products; ineffective disinfection measures; impossibility to restrict spread by wild animals; biosecurity measures totally ineffective.

D3‐2	*Vaccine availability for animals*	
	Score 0	
	Score 1	Very high: commercialized vaccine available on a global scale (worldwide).
	Score 2	High: local/monostrain vaccine available at a regional/national scale (not systematically available for a global fight plan).
	Score 3	Low: experimental vaccine, not commercialized to date; severe adverse reaction when applied; limited protective effect.
	Score 4	Very low: absence; no vaccine available on the market for use in the species considered in the study, no experimental vaccine either.

D3‐3	*Control of reservoir(s) and/or vector(s)*	
	Score 0	Null: no vector‐borne transmission and/or no reservoir(s) known to date.
	Score 1	Very high: effective. Limited reservoir(s) with limited geographical repartition, easy‐to‐identify; high scientific knowledge on vector(s)/reservoir(s); effective control measures.
	Score 2	High: limited reservoir(s)/vector(s) with limited geographical repartition; easy‐to‐identify, high scientific knowledge on vector(s)/reservoir(s); effective control measures but NOT applicable at a large scale; limited fighting measures.
	Score 3	Low: numerous reservoirs vectors identified with limited geographical repartition; hard to identify. Lack of scientific knowledge on vector(s)/reservoir(s). Control measures are poorly effective—resistances and/or negative impact on the environment.
	Score 4	Very low: numerous Vector(s)/reservoir(s) identified with wide geographic distribution; hard to identify, absence of scientific knowledge on vector(s)/reservoir(s); NO effective control measure against vector(s) (no active molecule, resistance to measures applied).

D3‐4	*Clinical detection of circulation of the pathogen in animals—e.g., difficulties for the farmers/veterinarians to report the disease or clinical signs not evident.*	
	Score 0	
	Score 1	Very high: disease is easily detected through clinical signs and farmers are aware of the disease and willing to notify as soon as possible.
	Score 2	High: disease is easily detected through clinical signs but farmers have neither sufficient knowledge/awareness nor interest to notify it.
	Score 3	Moderate: disease is not easily detected although the clinical signs and farmers have neither sufficient knowledge/awareness nor interest to notify.
	Score 4	Low: the infected animal does not show any pathognomonic clinical sign(s); the farmer is reluctant to declare/notify any abnormality.

D3‐5	*Methods to detect the virus in animals in your country (genome or virus that may be infectious)*	
	Score 0	
	Score 1	Very high: field test(s) available and easy to use, with highly discriminating sensitivity and specificity (including infectivity tests).
	Score 2	High: tests used in local/regional laboratories but not in the field.
	Score 3	Low: tests only used in *specialized* laboratories/national reference laboratory.
	Score 4	Very low: no detection methods available to date.

D3‐6	*Methods for detecting viral agent in vectors (e.g., Culicoides) in your country*	
	Score 0	
	Score 1	Very high: field test(s) available and easy to use, with highly discriminating sensitivity and specificity (including infectivity tests).
	Score 2	High: tests used in local/regional laboratories but not in the field.
	Score 3	Low: tests only used in *specialized* laboratories/national reference laboratory.
	Score 4	Very low: no detection methods available to date.

D3‐7	*Disease in animals is currently under surveillance overseas (WOAH, EU)*	
	Score 0	
	Score 1	Very high: generalized surveillance implemented by all EU Member States and worldwide surveillance.
	Score 2	High: surveillance of the pathogen only by EU Member States.
	Score 3	Low: surveillance only in some EU Member States (because they had cases of the disease) and only in some non‐EU countries (not a disease reported by any international organizations).
	Score 4	Very low: absence of surveillance of the pathogen in all EU member countries and worldwide.

D3‐8	*Experience on disease eradication experience in other countries and/or your country*	
	Score 0	
	Score 1	Very high: previous experience on eradication has been applied fast and successfully.
	Score 2	High: previous experience on eradicating the disease but with some setbacks in the process.
	Score 3	Low: knowledge on eradication procedures but eradication program never implemented in your country.
	Score 4	Very low: disease is endemic, pathogen impossible to eradicate or novel disease to eradicate.

*Note:* Number of drivers = 8, hence 80 points to be distributed within this domain for the intradomain weighting.

Table A4

**Table A4:** Domain D4. Farm/European characteristics.

D4‐1	*Type of farms/type of production: dairy/beef (cattle) production, sheep or goat production, single-species farms—one single animal species farmed (e.g., only cattle) or multispecies farms (farms with more than one species, e.g., goats and cattle on the same farm/land/premises).*	
	Score 0	
	Score 1	Negligible: the type of farm does not influence the incidence of animal disease in your country.
	Score 2	Low: the type of farm has a low influence on the incidence of animal disease in your country.
	Score 3	Moderate: the type of farm has a moderate influence on the incidence of animal disease in your country.
	Score 4	High: the type of farm has a high influence on the incidence of animal disease in your country.

D4‐2	*Type of farming. Extensive (small holders with a few animals) versus intensive farming*	
	Score 0	
	Score 1	Negligible: animal farm density has a negligible effect on the incidence of animal disease in your country.
	Score 2	Low: type of farming (extensive or intensive) has a low effect on the incidence of animal disease in your country.
	Score 3	Moderate: type of farming (extensive or intensive) has a moderate effect on the incidence of animal disease in your country.
	Score 4	High: type of farming (extensive or intensive) has a high effect on the incidence of animal disease in your country.

D4‐3	*Farm management practices (off-ground farming, treatment, feeding practices, etc.)*	
	Score 0	
	Score 1	Negligible: management practices have a negligible effect on the incidence of animal disease in your country.
	Score 2	Low: management practices have a low effect on the incidence of animal disease in your country.
	Score 3	Moderate: management practices have a moderate effect on the incidence of animal disease in your country.
	Score 4	High: management practices have a high effect on the incidence of animal disease in your country.

D4‐4	*The number of animals infected by EHDV in the herd (infection rate)*	
	Score 0	
	Score 1	Negligible: the number of animals infected by EHDV in farms has a negligible effect on the incidence of animal disease in your country.
	Score 2	Low: the number of animals infected by EHDV in farms has a low effect on the incidence of animal disease in your country.
	Score 3	Moderate: the number of animals infected by EHDV in farms has a moderate effect on the incidence of animal disease in your country.
	Score 4	High: the number of animals infected by EHDV in farms has a high effect on the incidence of animal disease in your country.

D4‐5	*The rural (farm)-wildlife interface*	
	Score 0	
	Score 1	Negligible: the disease has never (re)emerged from the farm/wildlife interface.
	Score 2	Low: the disease has a low probability to (re)emerge via the farm/wildlife interface. The disease has been known to (re)emerge from the wild bush but very rarely.
	Score 3	Moderate: the disease has a moderate probability to occur via the farm/wildlife interface. Barriers (natural or artificial) are needed to prevent disease incidence in livestock.
	Score 4	High: there is a high probability for the disease to (re)emerge via the farm/forest interface. Barriers (natural or artificial) separating farms from natural forests are ineffective.

*Note:* Number of drivers = 5, hence 50 points to be distributed within this domain for the intradomain weighting.

Table A5

**Table A5:** Domain D5. Global changes.

D5‐1	*Influence of rainfall on the abundance/survival of vector populations*	
	Score 0	
	Score 1	Negligible: abundance of vector populations is not influenced by increased rainfall.
	Score 2	Low: abundance of vector populations is slightly influenced by increased rainfall.
	Score 3	Moderate: abundance of vector populations is moderately influenced by increased rainfall.
	Score 4	High: abundance of vector populations is highly influenced by increased rainfall.

D5‐2	*Influence of humidity on the abundance/survival of vector populations*	
	Score 0	
	Score 1	Negligible: abundance of vector populations is not influenced by increased humidity.
	Score 2	Low: abundance of vector populations is slightly influenced by increased humidity.
	Score 3	Moderate: abundance of vector populations is moderately influenced by increased humidity.
	Score 4	High: abundance of vector populations is highly influenced by increased humidity.

D5‐3	*Influence of temperature on the abundance/survival of vector populations*	
	Score 0	
	Score 1	Negligible: abundance of vector populations is not influenced by increased temperature.
	Score 2	Low: abundance of vector populations is slightly influenced by increased temperature.
	Score 3	Moderate: abundance of vector populations is moderately influenced by increased temperature.
	Score 4	High: abundance of vector populations is highly influenced by increased temperature.

D5‐4	*Changes of landscape, e.g., landscape fragmentation, creation of barriers, landfill sites.*	
	Score 0	
	Score 1	Negligible: changes in landscape have a negligible effect on the (re)emergence/incidence of pathogen/disease.
	Score 2	Low: changes in landscape have a low effect on the (re)emergence/incidence of the disease/pathogen but need other factors (e.g., land use changes combined with higher winter temperatures).
	Score 3	Moderate: landscape changes increase the density of reservoir hosts or availability of vectors or increase the pathogen survival or increase the contact between animals and vectors. Empty land can also create an environment for disease‐carrying wildlife.
	Score 4	High: landscape changes are one of the main drivers for the pathogen or its vectors or the contact between animals and vectors.

*Note:* Number of drivers = 4, hence 40 points to be distributed within this domain for the intradomain weighting.

Table A6

**Table A6:** Domain D6. Wildlife.

D6‐1	*Potential role of zoos in the (re)emergence of the pathogen or increasing incidence of the disease in animals*	
	Score 0	Not applicable: the disease has not been reported in zoos.
	Score 1	Negligible: the disease can be present in zoo animals, but its transmission from zoo animals to other animal species has not been reported to date.
	Score 2	Low: the disease can enter a zoo (e.g., introduction of an infected exotic animal), but only accidental transmission of the disease from zoo animals to other animal species has been reported. Hence, zoos have a low effect on the (re)emergence of the disease or increasing incidence of the disease in animals.
	Score 3	Moderate: the disease can enter a zoo and be present in zoo animals, but it requires the implication of a vector (biological/mechanical) to be transmitted to other animal species. Therefore, zoos have a moderate effect on the (re)emergence of the disease or increasing incidence of the disease in animals.
	Score 4	High: the disease can be introduced to a zoo via an infected imported animal; zoo animals can carry the disease that can easily jump to other animal species.

D6‐2	*Increase of indigenous wild mammals in Europe and neighbouring countries*	
	Score 0	Not applicable: disease has not been reported in wildlife.
	Score 1	Negligible: the increase in indigenous mammal populations does not affect the risk of (re)emergence or increasing incidence of the disease in animals.
	Score 2	Low: the slight increase in indigenous mammal populations can slightly increase the probability of (re)emergence or increasing incidence of the disease in animals.
	Score 3	Moderate: the increase in wild mammal populations has been associated with a (re)emergence/increasing incidence of the disease in animals.
	Score 4	High: the increase in wild mammal populations is the only factor associated with outbreaks of the disease in animals.

D6‐3	*Increase in endemic/migrating populations of wild animals (e.g., reindeer and wolf)*	
	Score 0	Not applicable: wild/migrating animals are not a reservoir of the disease or responsible of the spread of the pathogen through vector transport.
	Score 1	Negligible: there is a negligible probability of disease (re)emergence/increasing incidence in animals because of increased populations of endemic/migrating wild animals.
	Score 2	Low: there is a low probability of disease (re)emergence/increasing incidence in animals and spread through increased populations of endemic/migrating wild animals. Disease has spread from the endemic/migrating wild animals but only accidentally or under exceptional circumstances.
	Score 3	Moderate: there is a moderate probability of the disease to be introduced and spread through increased populations of endemic/migrating wild animals.
	Score 4	High: there is a high probability for disease (re)emergence/increasing incidence in animals through increased populations of wild/migrating animals. These are hosts or reservoirs of the disease.

D6‐4	*Hunting activities: hunted animals can be brought back to where livestock are present*	
	Score 0	
	Score 0	Not applicable: pathogen or infected vector is not carried by hunted animals.
	Score 1	Negligible: the risk of disease/pathogen (re)emergence in livestock due to hunting activities is practically null.
	Score 2	Low: disease is present in hunted wildlife and only accidental (re)emergence or increasing incidence of animal disease have been reported in livestock because of hunting. The risk of the disease/pathogen to(re)emerge in livestock due to hunting activities is practically null.
	Score 3	Moderate: disease is present in hunted wildlife, but a certain control is established by the hunter.
	Score 4	High: disease is present in hunted wildlife and hunting is one of the main modes of transmission of the disease to livestock.

D6‐5	*Transboundary movements of terrestrial wildlife from other countries*	
	Score 0	Not applicable: pathogen or infected vector is not carried by terrestrial wildlife.
	Score 1	Negligible: (re)emergence of disease or disease increasing by terrestrial movements of wildlife has only been suspected but never confirmed.
	Score 2	Low: there is a low probability for the disease to (re)emerge and spread through transboundary movements of terrestrial wildlife.
	Score 3	Moderate: there is a moderate probability for the disease to (re)emerge and spread through transboundary movements of terrestrial wildlife.
	Score 4	High: there is a high probability for the disease to (re)emerge and spread through transboundary movements of terrestrial wildlife. They are hosts and may spread/carry the disease.

*Note:* Number of drivers = 5, hence 50 points to be distributed within this domain for the intradomain weighting.

Table A7

**Table A7:** Domain D7. Human activities.

D7‐1	*Human movements linked to tourism*	
	Score 0	
	Score 1	Negligible: tourism is a negligible driver on the emergence or re‐emergence or increasing animal disease incidence in the country of interest.
	Score 2	Low: tourism increase is a low driver of the (re)emergence or increase of animal disease incidence in the country of interest.
	Score 3	Moderate: tourism increase is a moderate driver for the (re)emergence or increase of animal disease incidence in the country of interest. Biosecurity measures are sufficient to prevent the entering of the pathogen.
	Score 4	High: tourist movement is a high driver on the (re)emergence or increase of animal disease incidence in the country of interest. Tourists are highly likely to bring the disease into your country in their belongings (e.g., infected vector) and biosecurity measures are insufficient to stop pathogen entry.

D7‐2	*Human immigration*	
	Score 0	
	Score 1	Negligible: immigration movements are a negligible driver of disease (re)emergence or of increasing incidence in animals.
	Score 2	Low: immigration movements are a low driver of disease (re)emergence or of increasing incidence in animals.
	Score 3	Moderate: immigration movements are a moderate driver of disease (re)emergence or increasing incidence in animals in the country of interest. Disease is highly likely to emerge using this route, but biosecurity measures are sufficient to avoid (re‐)emergence or increasing disease incidence in the country of interest.
	Score 4	High: immigration movements have a high effect as a driver on the (re)emergence or increasing incidence in animals in the country of interest. Disease is highly likely to emerge using this route as biosecurity measures are not sufficient to avoid (re‐)emergence or increasing disease incidence in the country of interest.

D7‐3	*Transport movements: more specifically commercial flights, commercial transport by ships, cars or military (excluding transport of live animals)*	
	Score 0	
	Score 1	Negligible: the role of commercial movements as a driver on the (re)emergence or increasing disease incidence in animals in your country is negligible.
	Score 2	Low: the role of commercial movements as a driver on the (re)emergence or increasing disease incidence in animals is low. It is easily preventable by implementing biosecurity measures.
	Score 3	Moderate: the role of commercial movements as a driver on the (re)emergence or increasing disease incidence in animals is moderate. Disease can be prevented if biosecurity measures are tightened.
	Score 4	High: the role of commercial movements as a driver on the (re)emergence or increasing incidence of the disease in animals is high. Disease is hard to control via the current biosecurity measures.

D7‐4	*Live animal transport vehicles*	
	Score 0	
	Score 1	Negligible: the role of live animal transport vehicles as a driver of (re)emergence or increasing incidence of the disease in animals is negligible.
	Score 2	Low: the role of live animal transport vehicles as a driver of (re)emergence or increasing incidence of the disease in animals is low.
	Score 3	Moderate: the role of live animal transport vehicles as a driver of (re)emergence or increasing incidence of the disease in animals is moderate.
	Score 4	High: the role of live animal transport vehicles as a driver of (re)emergence or increasing incidence of the disease in animals is high.

D7‐5	*Bioterrorism potential*	
	Score 0	
	Score 1	Negligible: the role of bioterrorism as a driver of disease (re)emergence or increasing incidence in animals is negligible; the pathogen is unavailable and/or difficult and has low spread potential.
	Score 2	Low: the role of bioterrorism as a driver of disease (re)emergence or increasing incidence in animals is low; the pathogen is available and easy to handle by professionals and labs but has a low spread potential.
	Score 3	Moderate: the role of bioterrorism as a driver of disease (re)emergence or increasing incidence in animals is moderate; the pathogen is available and easy to handle by professionals and labs and spreads rapidly.
	Score 4	High: the role of bioterrorism as a driver of disease (re)emergence or increasing incidence in animals is high; the pathogen is available and easy to handle by individuals and spreads rapidly.

D7‐6	*Inadvertent release of an exotic infectious agent from a containment facility, e.g., laboratory*	
	Score 0	
	Score 1	Negligible: the pathogen is not currently present in any laboratory.
	Score 2	Low: the pathogen is present in a containment facility but its release is very unlikely as it is very easily contained.
	Score 3	Moderate: the pathogen is present in a containment facility and its release can occur as not easily contained.
	Score 4	High: the pathogen is handled in a Risk 3 or 4 laboratory (BSL3 or BSL4) in the country. It can leave the facility if biosecurity measures are not implemented correctly and can easily spread to livestock.

*Note:* Number of drivers = 6, hence 60 points to be distributed within this domain for the intradomain weighting.

Table A8

**Table A8:** Domain D8. Economic and trade activities.

D8‐1	*Decrease of resources allocated to disease surveillance in animals and/or in the environment*	
	Score 0	
	Score 1	Negligible: resources allocated to disease surveillance have no effect on the (re)emergence/increasing incidence of the disease in your country. Disease has never been under surveillance.
	Score 2	Low: resources allocated to disease surveillance have a low effect on the (re)emergence/increasing incidence of the disease in your country. Disease has been under surveillance in the past and no change occurred after surveillance stopped.
	Score 3	Medium: resources allocated to disease surveillance have a moderate effect on the (re)emergence/increasing incidence of the disease in your country. Disease is under passive surveillance (reported only when observed) but with no need for increased surveillance.
	Score 4	High: resources allocated to the disease surveillance have a high effect on the (re)emergence/increasing incidence of the disease in your country. Disease needs to be under active and passive surveillance as (re)emergence/increasing incidence can easily occur. Therefore, if the surveillance decreases, it is very likely to (re)emerge.

D8‐2	*Modification of the disease status (i.e., reportable disease becoming not reportable) or change in the screening frequency due to a reduced national budget*	
	Score 0	
	Score 1	Negligible: modification of the disease status due to a reduced national budget has a negligible effect on the (re) emergence/increasing incidence of the disease in your country.
	Score 2	Low: modification of the disease status due to a reduced national budget has a low effect on the (re) emergence/increasing incidence of the disease in your country.
	Score 3	Moderate: modification of the disease status due to a reduced national budget has a moderate effect on the (re) emergence/increasing incidence of the disease in your country.
	Score 4	High: modification of the disease status due to a reduced national budget has a high effect on the (re) emergence/increasing incidence of the disease in your country.

D8‐3	*Decrease of resources allocated to the implementation of biosecurity measures at border controls (e.g., harbours or airports).*	
	Score 0	Endemic disease
	Score 1	Negligible: decreasing the resources allocated to the implementation of biosecurity measures has a negligible effect on the disease (re)emergence or incidence in your country. Disease has never been detected in the past in a harbour or in an airport.
	Score 2	Low: decreasing the resources allocated to the implementation of biosecurity measures has a low effect on the disease (re)emergence or incidence in your country. The introduction of the disease in other countries because of deficient biosecurity at border controls has been suspected.
	Score 3	Medium: decreasing the resources allocated to the implementation of biosecurity measures has a moderate effect on the disease (re)emergence or incidence in your country. The disease has been introduced in other countries because of deficient biosecurity at border controls.
	Score 4	High: decreasing the resources allocated to the implementation of biosecurity measures highly increases the risk of (re)emergence or increasing incidence of the disease in your country. In the past, the disease has been introduced in other countries AND in your country because of deficient biosecurity at border controls.

D8‐4	*Influence of illegal or legal movements of live animals (e.g., livestock) from neighbouring/European Union Member States (MS) on disease (re)emergence/increased incidence in your country*	
	Score 0	
	Score 1	Negligible: illegal or legal movements of live animals (e.g., livestock) from neighbouring/EU Member States have a negligible influence on the pathogen/disease (re)emergence/increasing incidence in your country.
	Score 2	Low: illegal or legal movements (e.g., livestock) from neighbouring/EU Member States have a low influence on the pathogen/disease (re)emergence/increasing incidence in your country.
	Score 3	Moderate: illegal or legal movements (e.g., livestock) from neighbouring/EU Member States have a moderate influence on the pathogen/disease (re)emergence/increasing incidence in your country.
	Score 4	High: illegal or legal movements (e.g., livestock) from neighbouring/EU Member States have a high influence on the pathogen/disease (re)emergence/increasing incidence in your country.

D8‐5	*Influence of (il)legal EU trade exchanges of food products (e.g., raw milk cheese products) on disease (re)emergence in your country*	
	Score 0	
	Score 1	Negligible: increased imports of food products such as raw meat/dairy products from European countries have a negligible influence on the pathogen/disease (re)emergence/increased incidence in your country.
	Score 2	Low: increased imports of food products such as raw meat/dairy products from European countries have a low influence on the pathogen/disease (re)emergence/increased incidence in your country.
	Score 3	Moderate: increased imports of food products such as raw meat/dairy products from European countries have a moderate influence on the pathogen/disease (re)emergence/increased incidence in your country.
	Score 4	High: increased imports of food products such as raw meat/dairy products from European countries have a high influence on the pathogen/disease (re)emergence/increased incidence in your country.

D8‐6	*Influence of increased illegal or legal imports of animal subproducts such as skin and edible products from neighbouring/European Union Member States (MS) on disease (re)emergence/increased incidence in your country*	
	Score 0	
	Score 1	Negligible: increased imports of animal subproducts such as skin and edible products from neighbouring/European Union Member States (MS) have a negligible influence on the pathogen/disease (re)emergence/increased incidence in your country.
	Score 2	Low: increased imports of animal subproducts such as skin and edible products from neighbouring/European Union Member States (MS) have a low influence on the pathogen/disease (re)emergence/increased incidence in your country.
	Score 3	Moderate: increased imports of animal subproducts such as skin and edible products from neighbouring/European Union Member States (MS) have a moderate influence on the pathogen/disease (re)emergence/increased incidence in your country.
	Score 4	High: increased imports of animal subproducts such as skin and edible products from neighbouring/European Union Member States (MS) have a high influence on the pathogen/disease (re)emergence/increased incidence in your country.

D8‐7	*Influence of increased illegal or legal imports of nonanimal products such as* tyres*, wood, furniture from EU Member States on the disease/pathogen (re)emergence/increased incidence in your country*	
	Score 0	
	Score 1	Negligible: illegal or legal movements of nonanimal products such as tyres, wood and furniture from EU Member States have a negligible influence on the pathogen/disease (re)emergence/increased incidence in your country.
	Score 2	Low: illegal or legal movements of nonanimal products such as tyres, wood and furniture from EU Member States have a low influence on the pathogen/disease (re)emergence/increased incidence in your country.
	Score 3	Moderate: illegal or legal movements of nonanimal products such as tyres, wood and furniture from EU Member States have a moderate influence on the pathogen/disease (re)emergence/increased incidence in your country.
	Score 4	High: illegal or legal movements of nonanimal products such as tyres, wood, furniture from EU Member States have a high influence on the pathogen/disease (re)emergence/increased incidence in your country.

D8‐8	*Influence of illegal or legal movements of live animals (e.g., livestock) from third countries on the disease/pathogen (re)emergence/increased incidence in your country*	
	Score 0	
	Score 1	Negligible: illegal or legal movements of live animals (e.g., livestock) from third countries have a negligible influence on the pathogen/disease for the disease/pathogen (re)emergence/increased incidence in your country.
	Score 2	Low: illegal or legal movements of live animals (e.g., livestock) from third countries have a low influence on the pathogen/disease for the disease/pathogen (re)emergence/increased incidence in your country.
	Score 3	Moderate: illegal or legal movements of live animals (e.g., livestock) from third countries have a moderate influence on the pathogen/disease for the disease/pathogen (re)emergence/increased incidence in your country.
	Score 4	High: illegal or legal movements of live animals (e.g., livestock) from third countries have a high influence on the pathogen/disease for the disease/pathogen (re)emergence/increased incidence in your country.

D8‐9	*Influence of (il)legal third countries trade exchanges of food products (dairy, raw products) on disease (re)emergence in your country*	
	Score 0	
	Score 1	Negligible: increased imports of food products such as raw meat products from third countries have a negligible influence on the pathogen/disease (re)emergence/increased incidence in your country.
	Score 2	Low: increased imports of food products such as raw meat products from third countries have a low influence on the pathogen/disease (re)emergence/increased incidence in your country
	Score 3	Moderate: increased imports of food products such as raw meat products from third countries have a moderate influence on the pathogen/disease (re)emergence/increased incidence in your country.
	Score 4	High: increased imports of food products such as raw meat products from third countries have a high influence on the pathogen/disease (re)emergence/increased incidence in your country.

D8‐10	*Influence of increased imports of animal subproducts (skin and edible products) from third countries, on disease/pathogen (re)emergence/increased incidence in your country*	
	Score 0	
	Score 1	Negligible: increased imports of animal subproducts (skin and edible products) from third countries have a negligible influence on the pathogen/disease (re)emergence/increased incidence in your country.
	Score 2	Low: Increased imports of animal subproducts (skin and edible products) from third countries have a low influence on the pathogen/disease (re)emergence/increased incidence in your country.
	Score 3	Moderate: increased imports of animal subproducts (skin and edible products) from third countries have a moderate influence on the pathogen/disease (re)emergence/increased incidence in your country.
	Score 4	High: increased imports of animal subproducts (skin and edible products) from third countries have a high influence on the pathogen/disease (re)emergence/increased incidence in your country.

D8‐11	*Influence of increased illegal or legal imports of nonanimal products (tyres, wood, furniture) from third countries, on the disease/pathogen (re)emergence/increased incidence in your country*	
	Score 0	
	Score 1	Negligible: increased illegal or legal imports of nonanimal products (tyres, wood, furniture) from third countries have a negligible influence on the pathogen/disease (re)emergence/increased incidence in your country.
	Score 2	Low: increased illegal or legal imports of nonanimal products (tyres, wood, furniture) from third countries have a low influence on the pathogen/disease (re)emergence/increased incidence in your country.
	Score 3	Moderate: increased illegal or legal imports of nonanimal products (tyres, wood, furniture) from third countries have a moderate influence on the pathogen/disease (re)emergence/increased incidence in your country.
	Score 4	High: increased illegal or legal imports of nonanimal products (tyres, wood, furniture) from third countries have a high influence on the pathogen/disease (re)emergence/increased incidence in your country.

*Note:* Number of drivers = 11, hence 110 points to be distributed within this domain for the intradomain weighting.

## Appendix A2: Profile of Experts Involved in the Elicitation of Knowledge in Alphabetical Order (*N* = 41).

Table A9

**Table A9:** Profile of experts.

ID	Name	Surname	Gender	ISO	Level	Employment	Competency	Keyword 1	Keyword 2	Keyword 3
1.	Alba‐Casals	Anna	F	SP	R	GI	PH; AH	Epidemiology	Surveillance	Veterinary science
2.	Badiola	Juan	M	SP	N; R; I	UN	PH; AH; FS; LD	Veterinary pathology	Animal health	Food safety
3.	Balenghien	Thomas	M	FR	N	RS	AH	Entomology	Transmission	Ecology
4.	Bayrou	Calixte	M	BE	N	UN	AH; LD	Cattle	Pathogens	Immunity
5.	Bertagnoli	Stéphane	M	FR	N	UN	AH	Virology	Vaccinology	Vectorology
6.	Cardinale	Eric	M	FR	I	RS	PH; AH; FS	Epidemiology of infectious diseases	Antimicrobial resistance	Zoonoses (including food safety)
7.	Casal	Jordi	M	SP	N	UN	AH	Risk analysis	Disease spread	Economics of disease
8.	Cassart	Dominique	F	BE	R	UN	PH; AH	Autopsy	Macroscopic and microscopic lesions	Differential diagnosis
9.	Dal Pozzo	Fabiana	F	BE	N	NGO	PH; AH	AMR	Biosecurity	Vector‐borne diseases
10.	De Clercq	Kris	M	Int.	I	PC	AH; LD	Exotic diseases	Laboratory diagnosis	Disease control
11.	De Regge	Nick	M	BE	N	GI	AH; LD	Virology	Vector‐borne diseases	Diagnostics
12.	Delooz	Laurent	M	BE	N; R	UN; DL	PH; AH; LD	Cattle abortion	Infectious disease	Epidemiology
13.	England	Marion	F	UK	N	RS	AG; EC	Entomology	Vector ecology	Vector surveillance
14.	Filippitzi	Maria Elena	F	GR	N; I	UN	PH; AH	Veterinary epidemiology	Risk analysis	Antimicrobial resistance
15.	Garigliany	Mutien	M	BE	R	UN	AH; LD	Pathology	Host‐pathogen interactions	Virus evolution
16.	Garin	Emmanuel	M	FR	N	NGO	PH; AH	Epidemiology	Diseases management	Epidemiosurveillance network
17.	Garros	Claire	F	FR	N; R; I	RS	AH	Medical & veterinary entomology	Surveillance	Bioecology & taxonomy
18.	Gisbert	Philippe	M	Int.	I	PC	PH; AH; LD	Antibiotics/AMR	Laboratory diagnostics	Vaccines
19.	Gortazar	Christian	M	SP	N	UN	AH; EC	Wildlife diseases	Control of shared infections	Wildlife management
20.	Grisot	Lionel	M	FR	N; R	RS; PC	PH; AH; FS	Veterinary medicine	Animal health	one health
21.	Guyot	Hugues	M	BE	N; R; I	UN	AH	Bovine herd health management	Internal medicine, diagnosis	Ruminants physiology and nutrition
22.	Haddad	Nadia	F	FR	N	UN; RS	PH; AH	Zoonoses	Tick‐borne pathogens	Epidemiology
23.	Hoffmann	Bernd	M	DE	N; I	GI; DL	AH; LD	National reference laboratory	Diagnostics	Animal studies
24.	Hooyberghs	Jozef	M	BE	N	GI	AH	Animal health legislation	Control and eradication programmes	Animal disease management
25.	Humblet	Marie‐France	F	BE	R	UN	AH	Animal biosecurity	Infection control	Epidemiology
26.	Jiménez‐Cabello	Luis	M	SP	N	RS	AH	Orbivirus	Vaccine	Animal health
27.	Linden	Annick	F	BE	N	UN	PH; AH; LD	Wildlife diseases	Interface livestock	Zoonoses
28.	Martinelle	Ludovic	M	BE	N; R; I	UN	PH; AH	Epidemiology	Emerging diseases	Host–pathogen interaction
29.	Mathis	Alexander	M	CH	N	UN	PH; AH; LD	Arthropod vectors	Control	Parasitology
30.	Mauroy	Axel	M	BE	N	GI	AH	Risk assessment	Emerging risk	Risk management
31.	Meurens	François	M	FR	I	UN	AH	Host/pathogen interactions	Mucosal immunology	Veterinary microbiology
32.	Millemann	Yves	M	FR	N; I	UN	PH; AH	Cattle medicine	Resistance to antibiotics	Salmonella
33.	Ortego	Javier	M	SP	N	RS	AH	Orbivirus	Vaccines	Animal health
34.	Petit	Etienne	M	FR	R	SR	AH	Control of diseases	Epidemiology	Diagnostic tests
35.	Ruiz‐Fons	Francisco	M	SP	N; R; I	RS	PH; AH	Vector‐borne diseases	Wildlife	Epidemiology
36.	Savini	Giovanni	M	IT	N; R; I	GI; RS; DL	PH; AH; LD	Orbiviruses	Flaviviruses	Vector‐borne diseases
37.	Thiry	Etienne	M	BE	N	UN	AH	Viral diseases	Animal infectious diseases	Qualitative risk analysis
38.	Valarcher	Jean François	M	SE	N; R; I	UN	AH	Infectiology	Virus	Ruminants
39.	van Rijn	Piet A.	M	NL	N; I	GI; UN; RS	AH; LD	Diagnostics	Vaccine development	Molecular virology
40.	Zanella	Gina	F	FR	N	RS	AH	Epidemiology	Infectious diseases	Large domestic animals
41.	Zientara	Stéphan	M	FR	N; R; I	GI; RS; DL	PH; AH; LD	Virology	Animal health	Microbiology

*Note:* ISO = Standard defining codes for the names of countries and with Int. for international. Keywords: Maximum three and in decreasing order of importance. Level: N = National, R = Regional, I = International.

Abbreviations: AH = Animal health, DL = Diagnostic laboratory, EC = Ecology, EH = Environmental health, FS = Food safety, GI = Government institution, LD = Laboratory diagnostics, NGO = Nonprofit organization, PC = Private company, PH = Public health, RS = Research/scientific institution, UN = University.

## Appendix A3: Guidance Letter for the Expert Elicitation

**Ranking criteria of epizootic haemorrhagic disease (re-) emergence or increasing incidence in animals, in Europe**
**(Experts’ opinion)**
Dear expert, dear colleague,
This special request is related to the (re‐)emergence or increasing incidence of the epizootic haemorrhagic disease (EHD) in animals, in Europe.
A driver is defined as a factor that has the potential to directly or indirectly precipitate (‘drive’) or lead to the (re‐)emergence or increasing incidence of a disease.
The objective of the present study is to better understand the drivers (i.e., influencing factors) of (re‐)emergence or increasing incidence of EHD in animals, in Europe. The questionnaire was prepared in order to present different criteria of interest and summarized through 51 drivers. For each driver, scores are proposed with a corresponding definition. Drivers are grouped per category (*N* = 8), each category corresponding to one spreadsheet. After scoring, the weight of each driver in a specific category and in each category is calculated.
**Objective of the questionnaire**
We would like your opinion as an expert on the drivers of (re‐)emergence or increasing incidence of EHD in animals, in Europe.
Hence, to answer of the question: *‘What is the influence of different drivers on the (re)emergence or increasing incidence of EHD in animals, in Europe?’*
**How to fill the questionnaire**
In the attached excel questionnaire, there are **11 spreadsheets**. The first one summarises your expertise. The next 8 correspond to the 8 categories of drivers. The tenth spreadsheet corresponds to the intra‐category weighing and the last one to the level of uncertainty, per category of drivers.
1. Expert information
2. Pathogen characteristics: 9 criteria
3. Distance to Europe and your country: 3 criteria
4. Ability to monitor, treat and control the disease: 8 criteria
5. Farm/European characteristics: 5 criteria
6. Global change: 4 criteria
7. Wildlife interface: 6 criteria
8. Human activities: 6 criteria
9. Economy and trade activities: 11 criteria
10. Intra‐category weighing
11. Level of uncertainty per domain of criteria
**Actions required**
1. **Score and balance each driver within each category of drivers:**
  - Please give a score according to your estimated importance of each driver in the (re)‐emergence or increasing incidence of EHD in animals, in Europe.
  - After scoring, please balance each driver for each category of drivers. Balancing the criteria will rely on the distribution of points between the different criteria in each category. The total number of points to be distributed among the drivers is specified for each category (each spreadsheet). For example, in the category ‘pathogen characteristics’: a total of 90 points must be distributed => for ‘distance to Europe and your country’, a total of 30 points must be distributed. If a driver has no importance in comparison with others, no points are allocated (zero).
2. **Intra-category weighing**: The next step of the process will consist of distributing 100 points between the **8 categories** of criteria (pathogen characteristics, distance from outbreaks, etc.). This step is on the 10th spreadsheet. The distribution will illustrate your perception of the influence of each category of drivers.
3. **Level of uncertainty**: in the last spreadsheet, give an overall level of uncertainty for each category of drivers using a scale from 0 (minimal uncertainty in my scoring) to 100 (maximum uncertainty in my scoring).
As an expert in the field, your collaboration is essential and will help us a lot for the good course of this project.
Thank you in advance for your collaboration and for the time spent in filling the file **before November 30, 2023**.
**Each expert who contributes to the study (i.e., questionnaire fully completed in due time) will be added as an expert consulted in the expected publication on the results of the elicitation.**
For any question, please contact me: claude.saegerman@uliege.be
Kind regards,
**Professor Claude Saegerman**
Research unit in epidemiology and risk
Analysis applied to veterinary sciences
Fundamental and applied research for animal & health
Department of infectious and parasitic diseases
Faculty of veterinary medicine
University of Liège
Quartier Vallée 2
Avenue de Cureghem 6, B43a, 3th floor
4000 Liège Sart‐Tilman
**Belgium**
Tél: +32(0)43664579
E‐mail: claude.saegerman@uliege.be

## Appendix A4: Details of the Methodology for This Expert Elicitation

1. Questionnaire design
A questionnaire was developed to determine the main drivers of the observed spread of EHD in Europe. A driver was defined as a factor that has the potential to directly or indirectly precipitate (‘drive’) or lead to increasing cases of EHD in Europe [56]. A former questionnaire created to rank drivers of emergence of animal and zoonotic diseases [56–59] was adjusted to capture specific possible drivers for EHD. Overall, 51 drivers were recognized and categorized into eight different domains (Appendix A1). The domains (D) were (D01) the disease’s/pathogen’s characteristics (*N* = 9 drivers); (D02) the distance to Europe and the expert’s country (spatially‐temporally scales) (*N* = 3 drivers); (D03) the capacity to investigate, treat and control the disease (*N* = 8 drivers); (D04) the characteristics of European farms (*N* = 5 drivers); (D05) global change (*N* = 4 drivers); (D06) the interaction with wildlife (*N* = 5 drivers); (D07) human activity (*N* = 6 drivers); and (D08) economic and trade activities (*N* = 11 drivers). They were organized in an Excel file that was formatted for Microsoft Excel (Microsoft, Redmond, WA, 2016) with one table per domain, each domain having its own drivers. Each driver had a description that was specific to their definition, which could have a range of 0–4 (i.e., five modalities) or range of 1–4 (i.e., four modalities) and a weight point for the intra driver. A spreadsheet was incorporated that listed the eight domains along with their respective weights in the inter domain.
2. Expert elicitation to assess drivers of observed increasing cases of epizootic haemorrhagic disease in Europe
An expert elicitation of knowledge was conducted, which consisted of gathering the opinion of people with recognized scientific expertise (indicated by at least one publication as first or co‐author) in virology, laboratory diagnostic, epidemiology, ecology, entomology and wildlife and/or experience on EHD/EHDV in Europe (Appendix A2). The European experts participating in this study had diverse backgrounds and occupations, including government institutions, universities, research/scientific institutions, diagnostic laboratories and private companies. They represented various sectors related to EHD/EHDV, such as animal health, environment, laboratory diagnostics and ecology. Special consideration was given to ensure representation from all disciplines associated with the different driver domains. The distribution of professional expertise years followed a normal pattern (Shapiro–Wilk test; *p* value = 0.91), with an average of 25.5 years (standard error: 9.6). To provide guidance, a letter of explanation was included along with the questionnaire (see Appendix A3). Each expert was individually contacted and responded in their personal capacity, separate from their affiliations with their respective institutions.
The data generated by the elicitation were based on scores provided by the experts to capture the degree of variability in experts’ knowledge. The process of elicitation spanned a duration of 2 months (October–November 2023).
3. Scoring, weighting system and level of uncertainty
The elicited experts were asked to provide four types of information. Firstly, they were asked to score the drivers (as established in Appendix A1). For each driver, the higher the score, the higher the likelihood of contributing to the observed increasing EHD incidence in Europe is. Secondly, experts were requested to weight each driver within a specific domain (intradomain weight). This relative weight was determined using the Las Vegas technique [60]. In summary, a set of points were assigned to experts and these were divided among drivers based on the drivers’ relative importance within the given domain (proportional piling). If all the drivers of a given domain were considered equivalent by the experts, each of them would have received the same score. Thirdly, the relative importance of each domain was subsequently weighted by experts (inter‐domain weight). Finally, to reduce the time required for each expert to complete the questionnaire, the degree of uncertainty was queried at the domain level rather than for each driver, using a scale from 0 (minimum uncertainty in the scoring) to 100 (highest uncertainty in the scoring).
4. Calculation of an overall weighted score for each driver and ranking process
To obtain the overall score per driver, an aggregation method that combined the two types of weighting (i.e., the intra and interdomain) was used. First, the driver score (coefficients attributed by experts) was standardized by dividing it by the number of possibilities (i.e., modalities). Indeed, some drivers were allocated coefficients from 0 to 4 (five possibilities) and others from 1 to 4 (four possibilities). Afterwards, this standardized score was multiplied by the intradomain weight and the interdomain weight, as given by the expert. These results led to an overall weighted score for each driver and per expert:
  (A.1)
  OWSDri=SDri×WDri×WDoj.
In this formula, OWSDr*i* is the overall weighted score for a specific driver *i*; SDr*i* is the standardized score for a specific driver *i*; WDr*i* is the intradomain weight for a specific driver *i*; and WDo*j* is the interdomain weight for a specific driver *i* included in a specific domain *j*. Furthermore, all drivers were ranked based on the median overall weighted score obtained for each driver and considering the answers of all experts who answered the questionnaire. The statistical difference of the median, depending on the specific driver or the group of drivers considered, was assessed through a nonparametric Kruskal–Wallis equality‐of‐populations rank test and median regression analysis (Stata SE 14.2; StataCorp, College Station, Texas, USA).
5. Cluster analysis
A cluster analysis was carried out using a regression tree analysis (Salford Predictive Modeler®, Version 8.2, Salford Systems, San Diego, California, USA). A continuous variable, the median overall weighted score (median OWSDri) was used to obtain groups of drivers with minimal within‐group variance and with comparable likelihood to play a role in the observed increasing EHD incidence in Europe. In addition, the statistical difference between medians after grouping drivers in clusters was assessed using a nonparametric Kruskal–Wallis equality‐of‐populations rank test and a median regression analysis. Indeed, each driver was characterized by a median (based on all experts’ answers), and then, drivers were grouped. The test allowed the identification of potentially significant differences between groups for driver medians following clustering.
6. Sensitivity analysis to test the robustness of the expert elicitation
To identify whether the ranking of drivers of the observed increasing EHD incidence in Europe was influenced by the choice of experts or the country of origin of experts, a sensitivity analysis was performed. This analysis was conducted in two parts: first, comparing 10 bootstraps (a random selection of 30 experts out of 41), and second, comparing the drivers’ rankings from all the experts elicited as reference with those from Belgium (*N* = 13), France (*N* = 12) and other European countries (*N* = 16). The difference between these rankings was tested using the Pearson correlation coefficient. If this coefficient was close to 1 and *p* value less than 0.05, the correlation between the two rankings of drivers tested was considered significant.
